# Superior adsorption performance of citrate modified graphene oxide as nano material for removal organic and inorganic pollutants from aqueous solution

**DOI:** 10.1038/s41598-022-13111-6

**Published:** 2022-06-02

**Authors:** A. I. Abd-Elhamid, E. M. Abu Elgoud, Sh. Sh. Emam, H. F. Aly

**Affiliations:** 1 Composites and Nanostructured Materials Research Department, Advanced Technology and New Materials Research Institute (ATNMRI), City of Scientific Research and Technological Applications (SRTA-City), New Borg Al-Arab, 21934 Alexandria Egypt; 2 Hot Laboratories Center, Egyptian Atomic Energy Authority, Cairo, 13759 Egypt

**Keywords:** Pollution remediation, Graphene, Pollution remediation

## Abstract

This work addressed one step preparation method to form a novel nano material composite of graphene oxide nanosheet (GO) functionalized with low-cost tri-sodium citrate (C), using, teteraethylorthosilicate (TEOS) as a cross-linker. The prepared composite (GO–C) was characterized using various advanced techniques. Among these techniques, the TGA provided interesting information concerning the functionalization process. Within this process, the (–OH) groups that located at the GO-surface were consumed in the modification process which leads to increase the thermal stability of the resulted composite. Cationic organic methylene blue (MB) and crystal violet (CV), and inorganic copper (Cu^2+^) and cobalt (Co^2+^) pollutants were displayed as a model to assess their removal performance by the developed composite (GO–C) from aqueous solution, through batch technique. According to Langmuir isotherm the GO–C present an excellent adsorption capacity for MB (222.22 mg g^−1^), CV (270.27 mg g^−1^), Cu^2+^ (163.4 mg g^−1^) and Co^2+^ (145.35 mg g^−1^) which were more than the adsorption capacities found in literature. Additionally, the regenerated composite presents higher removal ability than the original composite.

## Introduction

Recently, one vital interest of environmental pollution is water desecration by different harm materials such as heavy metal ions and organic matters^1^. Among these organic substance, organic dyes and heavy elements that drained form several industries during their process^2^. This exemplified by paints, textile, cosmetics, leather, food. Discharge of the effluents resulted from these activities into the environments without proper management, will cause a great environmental defect^3^. Where, in aquatic environment the dye will appear even at low concentration which will retard the sunlight penetration required for the growth of aquatic plants, consequently, the aquatic life will be destroy. Therefore, the human will lose one of the most food sources, moreover, the accumulation of the dye in the water may become a health threat as they are toxic, recalcitrant, mutagenic, and carcinogenic^4^.

Another very vital pollutant are heavy metals, where the contact of the humans with such pollutant may cause a major health threat, this is due to its toxicity and carcinogenic influence^5^. These pollutants are not only cause a water contamination, but they are also cause a life risk^6^. Examples for the most harmful of the heavy metals involve Pb^2+^, Cd^2+^, As^2+^, Cu^2+^, Co^2+^, Ni^2+^, As^3+^, and Cr^7+^ and etc^7^. Moreover, several of the metal ion can even make a defect in the humans’ central nervous system and kidneys. Consequently, the removal of these pollutants from the wastewater attracts a high attention. Numerous techniques have been examined for pollutant removal. Adsorption strategy consider the most applicable technique applied for elimination of contaminates from wastewater. This is attributed to, its ease of operation, availability of adsorbent, case do not require complicated device and possibility of scale up. In this regard, the selection of suitable adsorbents is of very importance for achieving the appropriated results. Graphene oxide (GO) is intensively used in the last decade as a highly efficient adsorbent attributed to its high surface area and large number of the active sites. GO displayed as a 2D single layer adsorbent, with single C-atom thickness. Various oxygenated function groups (–OH and C–O–C) decorated its basal plane and (–COOH) located at the layer edge. These activated function groups consider as available negative active site that can form a complex with the cationic pollutants. In order to improve the adsorption efficiency of GO, numerous studies were investigated to liable GO in another constructures such as; graphene oxide (GO), sodium alginate (SA) and hydroxyethyl cellulose (HEC) (SA-HEC/GO bio-adsorbent hydrogel beads^8^, graphene oxide/hollow mesoporous silica (GO–HMS) composite^9^, sodium alginate/gelatin/graphene oxide (SGGO) nanocomposite^10^, polyaniline/graphene oxide (PANI/GO) nanocomposite ^11^, graphene oxide-chitosan-EDTA (GO–EDTA–CS) nanocomposite^12^, polyethyleneimine (PEI) modified (GO) to form (GO/PEI) sponge^13^, decoration of GO with zinc oxide nanoparticles (ZnO) (GO/ZnO) ^14^, graphene oxide (GO) modified with isocyanate (MDI), subsequently, (EDTA) (EDTA/MDI/GO) composite^15^, modification of graphene oxide (GO) nanosheets with magnetic particles of nickel ferrite (NiFe_2_O_4_) and followed by immobilizing glutathione (GSH) to fabrication of GSH-NiFe_2_O_4_/GO nanocomposite^16^ and Amino-modified graphene oxide (GONH_2_)^17^. Most of previous studies required expensive chemical, multi-preparation steps, low adsorption capacities, delayed equilibrium period, see Table 1.

**Table 1:** GO-based adsorbents for removal of organic and inorganic pollutants.

Adsorbent	Pollutant	Time	q_e_ (mg g^−1^)	Reusability, cycle	%R, after reuse	Activity reduction during the reuse (%)	Year	Refs.
SA-HEC/GO	CV	240 min	312	6	79.4	–	2022	^8^
GO/HMS	MB	120 min	476.19	5	85.3	–	2022	^9^
SGGO	CV MB EBT	180 min	255.12 175.34 115.42	7	68	–	2020	^10^
PANI/GO	MB	270 min	14.2	6	–	–	2020	^11^
GO–EDTA–CS	Hg^2^^+^ Cu^2^^+^ MB CV	120 min 120 min 180 min 180 min	324 ± 3.30 130 ± 2.80 141 ± 6.60 121 ± 3.50	7	– – – –	7.5	2022	^12^
GO/PEI	Cu^2+^	24 h	150.9	5	–	< 5	2022	^13^
GO/ZnO	Cu^2+^ Al^3+^	60 min	33.5 19.9	–	–	–	2020	^14^
EDTA/MDI/GO	Cu^2+^	180 min	254.2 ± 10.4	8	78	–	2018	^15^
GSH-NiFe_2_O_4_/GO	Cu^2+^ Pb^2+^ Hg^2+^	60 min	266.22 204.06 272.94	6	77.5–81	– –	2020	^16^
GONH_2_	Cr^6+^ Cu^2+^ Pb^2+^ Cd^2+^	4 h	280.11 26.25 71.89 10.04	– – – –	– – – –	– – – –	2020	^17^
GO–C	MB CV Cu^2+^ Co^2+^	5 min 7 min 1 min 1 min	222.22 270.27 163.40 145.35	5	~ 96.0 ~ 96.0 ~ 95.7 ~ 95.7			This study

Carboxyl groups (O=C–OH) are consisted from two functional groups hydroxyl (–OH) and carbonyl (C=O) which were bounded with the same C-atom. This unique combination between the two groups (–OH and C=O) create polar, high electronegative, weak acid group. At deprotonation, the carboxylate (COO^−^) anion was stabilized by resonance over the two O-atoms. This enables the carboxyl groups to form efficient complex with the cationic pollutants. Several GO carboxylation strategies were investigated, such as using of hydrobromic acid/oxalic acid^18,19^, and NaOH/chloroacetic acid^20–26^. Citric acid molecule is an ecofriendly, available and attractive complexing agent, that consists of one (–OH) group & one (–COOH) group at α-position and two (–COOH) groups at β-position, these groups can provide 7 (O-donor) sites for chelating the cationic species. These donor centers can localize around the cation ion in diverse arrangements as a complexing and bridging spacer^27^.

Here in, we study facile, single and environmentally modification step of the GO with low-cost, available and efficient chelating acid sodium salt (tri-sodium citrate) and using tetraethylorthosilicate (TOES) to form a bridge among the GO and citrate molecule. The modified material (GO–C) was characterized by various techniques; SEM, OM, FTIR, Raman and EDS. Cationic pollutants such as organic (MB, CV) and inorganic (Cu^2+^, Co^2+^) were used as models to examine the adsorptive efficiency of the prepared material.

## Experiment

### Preparation of GO and GO–C composite

**Figure 1:**
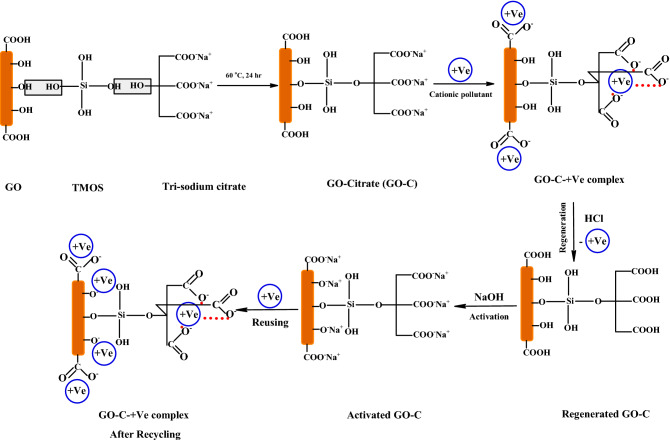
GO–C composite preparation, adsorption and regeneration/reused steps.

GO was prepared as investigated by our previous work^28^. The GO–C was simply prepared through one-put mixing process. Briefly, tri-sodium citrate (10 g) was added to 800 ml distill water and stirred variously until complete dissolution. To this solution, 150 mg GO were added and the stirring continued to form homogeneous suspension of the GO (solution a). In another beaker, 5.0 ml TMOS were added to 50 ml ethanol (solution b). Consequently, (solution b) added dropwise to the (solution a), the temperature of the final solution raised up to 60 °C and left on the stirrer for 24 h. The solid was filtrated and washed several times with distilled water and stored for further use. Figure 1, present a schematic illustration of GO–C composite preparation, adsorption and regeneration/reused steps.

## Results and discussion

### Characterization of GO–C

#### TEM, SEM and OM analysis

**Figure 2:**
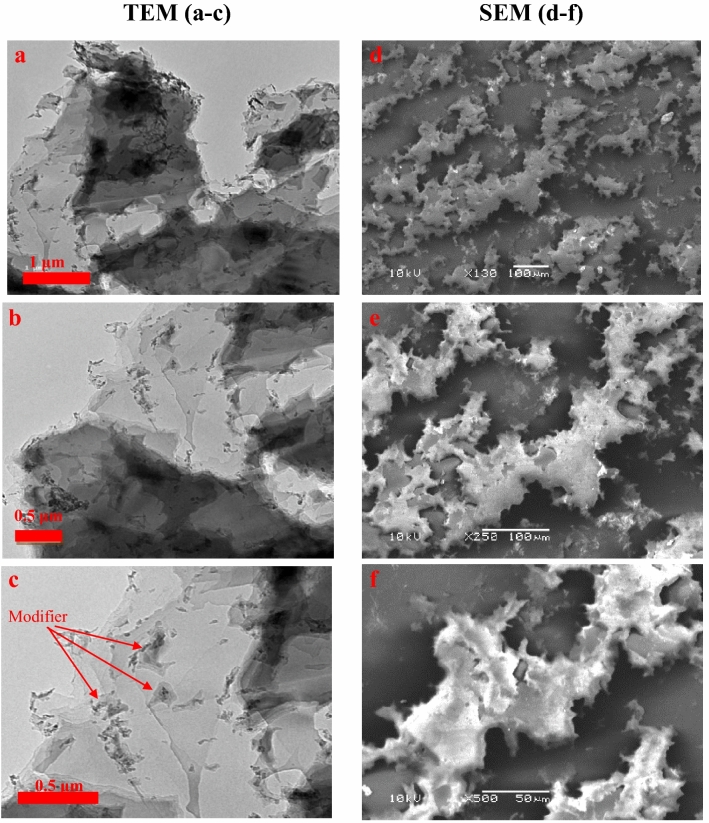
TEM (**a**–**c**, left) and SEM (**d**–**f**, right) of GO–C composite.

The TEM images of the GO–Cit composites recorded at various magnifications, explored that the GO–Cit composed from wrinkles and completely separated flattened nano-sheet like with tiny particles uniformly decorated its surface, as shown in Fig. 2a–c. This structure makes the surface function groups completely exposed to the adsorbed species, which enhance the adsorption ability and minimize the time required to achieve the equilibrium.

The SEM images of the prepared GO–C, before adsorption is given in Fig. 2d–f and after adsorption of methylene blue, GO–C–MB, and crystal violet, GO–C–CV is presented in Fig. S1. The images were magnified in the range 130×–500×. The morphology of the GO–C before and after the dye adsorption process was not highly differ and can prove the stability of the composite. The GO–C, GO–C–MB and GO–C–CV look like as a flaky layered structure decorated with precipitated material, which may be referred to the modifier, see Fig. 2e,f. These precipitated materials become more visible after the adsorption of the dye, which indicated the interaction between the dye and the carboxylate active groups, see Fig. S1.

The optical microscope (OM) was found as an efficient tool for characterization of graphitic materials. The GO–C, GO–C–MB and GO–C–CV were observed under the optical microscope at magnification range (5×, 10× and 20×), as shown in Fig. S2. In our previous work^28^ the GO appeared under the optical microscope as a smooth yellow layer. Herein, the GO–C appear as light-yellow colored areas, this color variation may be caused due to the modification process, as shown in Fig. S2. After mixing of GO–C and the dye (MB and CV) the dye color (blue for MB) and (violet for CV) homogenously distributed over the GO–C layer which demonstrated that these areas were involved on the active sites responsible for adsorption of the dye species, and these active sites uniformly distributed over the GO–C layer as presented in Fig. S2.

#### FT-IR spectrum

**Figure 3:**
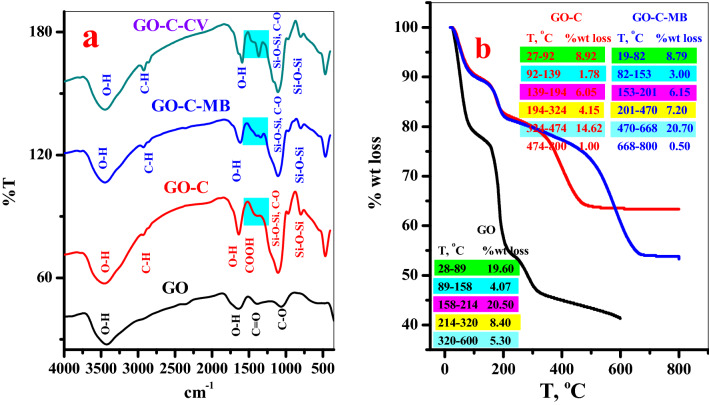
(**a**) FT-IR spectraGO, GO–C composite, GO–C–MB complex and GO–C–CV complex and (**b**) TGA diagram of GO, GO–C composite and GO–C–MB complex.

The chemical structure of the GO, GO–C, GO–C–MB and GO–C–CV samples were explored using the FTIR spectrum, Fig. 3a. The GO shows typical bands obtained in the previous study^28^; band at 3433 cm^−1^ (O–H stretching), 1635 cm^−1^(O–H bending), 1398 cm^−1^(COOH) and 1033 cm^−1^(C–O stretching)^29^. The GO–C present bands at 3455 cm^−1^(O–H stretching), 2931 cm^−1^(C–H stretching of aliphatic CH_2_)^30^, 1637 cm^−1^ (O–H bending), 1381 cm^−1^ (COOH)^21^, 11061 cm^−1^(Si–O–Si asymmetric stretching vibrations)^31^, 797 cm^−1^(bending vibrations of Si–O–Si)^31^. GO–C–MB and GO–C–CV shows shifts in the peaks at 3447 cm^−1^, 2927 cm^−1^, 1617 cm^−1^, 1394 cm^−1^ (for GO–C–MB) and 3447 cm^−1^, 2923 cm^−1^, 1591 cm^−1^, 1366 cm^−1^ (for GO–C–CV) which indicated successful interaction between the adsorbent and the dyes.

#### Raman spectra

Raman spectroscopy is exceedingly employed to explore the structure of the carboneous material. It is observed that, with increase the disorderly of the graphite, the bands at 1352 cm^−1^ (D-band) and 1598 cm^−1^ (G-band)^28^ become more-broader. Additionally, I_D_/I_G_, was employed as the sp^2^/sp^3^ ratio of the hybrid carbon^32^. Moreover, I_D_/I_G_ also express as the defects caused by the oxygen functional groups that linked to carbon skeleton and more, indicative of the integrity of the aromatic structure. The Raman spectra of the analyzed samples GO, GO–C, GO–C–MB and GO–C–CV, are demonstrated in Fig. S3. The locations of D and G-band, I_D_/I_G_ ratios and FWHMs are summarized in Table S1. As been noted from the Fig. S3, the I_D_/I_G_ ratio of the GO decrease after the modification process which indicate that the functionalization process may be repair of some defects located in the GO surface. After adsorption process the I_D_/I_G_ ratio tend to increase again which indicate consumption of the modifier in the adsorption of the dye.

#### TGA analysis

The thermo-gravimetric analysis is a useful technique to follow up the loss of the sample weight with further increase in the temperature. The thermal decomposition of GO, GO–C composite and GO–C–MB complex was carried out, as shown in Fig. 3b. And the decomposition temperature range, %wt loss and related degradable species was listed in Table S2. The GO decomposes over four stages similar to the previous work^28^, as indicated the surface moisture evaporated at temperature range, 28–89 °C, adsorbed water dehydration, 88–158 °C, pyrolysis of oxygenated function groups –OH and C–O–C, 158–215 °C, and finally, gravitation of –COOH, 215–320 °C^23^, as shown in Fig. 3b.

On the other hand, the thermograph of the GO–C present six decomposition stages, as explored in Fig. 3b. As pointed from the thermogram of the GO–C, Fig. 3b, the functionalization process leading to reduce the amount of the bounded surface water (Table S2). This is may be attributed to, most the hydroxyl (–OH) localized on the GO surface (which were responsible on the interaction with surrounding moisture via H-bonding) were contributed in the modification process as suggested (see Fig. 1). This can be noted from the thermograph (Fig. 3b) and the Table S2, where the GO loss 19.6% from its weight in first degradation stage, 28–89 °C, which crossponding to liberation of adsorbed surface water molecules. Followed by the main decomposition step (89–158 °C) which related to the pyrolysis of GO surface (OH) groups (weight loss = 20.50%). Whereas, in case of the GO–C, the amount of liberated moisture was evaluated (8.92%, 27–92 °C) followed by the second pyrolysis step which recorded a very low weight loss (1.78%, 92–139 °C), see (Fig. 3b) and Table S2. This may be clarifying that, the OH groups decorated the GO surface were shared in the modification process. Moreover, the GO–C provided high thermal stability than the GO, where, the GO–C possessed the main degradation stage in rang (324–474 °C) with 14.05% weight loss. This obtained result good agreement with literature^23^.

Further, by mixing of the GO–C with the dye solution, the dye species will be adsorbed on the different function groups (–OH, –COOH and –COONa) to form (GO–C–MB complex). This behavior may be resulted in covering GO–C with the dye species and isolated the composite far from the temperature effect leading to the increase of the thermal stability of the GO–C–MB, refer Fig. 3b and Table S2. Therefore, the GO–C–MB show the main thermal decomposition step in range (470–668 °C) with weight loss ratio 20.70%.

#### Energy-dispersive X-ray spectroscopy (ESD) analysis

**Figure 4:**
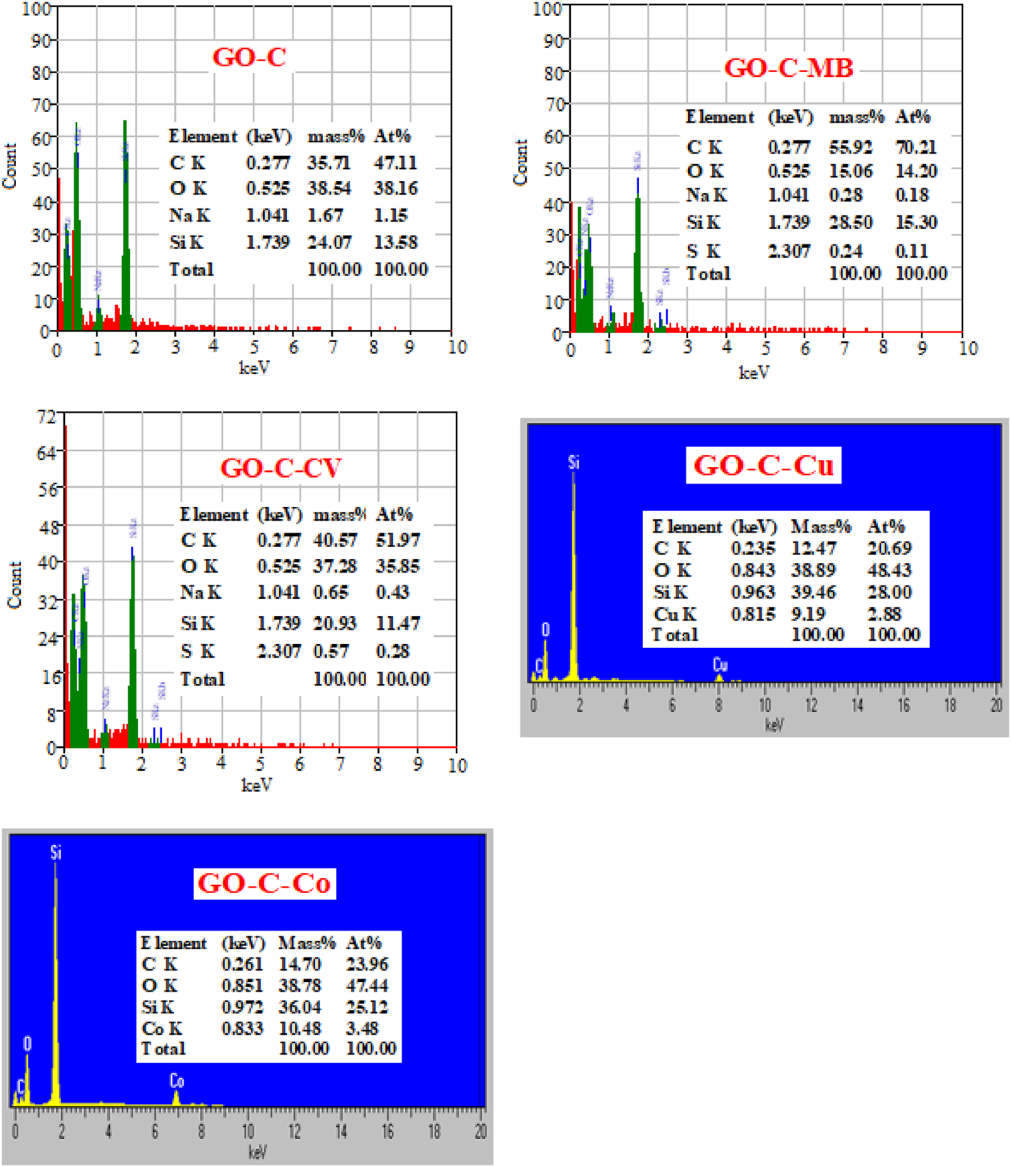
EDS analysis for GO–C composite, GO–C–MB complex, GO–C–CV complex, GO–C–Co complex and GO–C–Cu complex.

The importance of the EDS analysis is demonstrating on the elemental composition of the fabricated material. Graphene oxide is a carbonaceous material mainly composed of C and O-atoms. Herein, we modified the GO with tri-sodium citrate and using tetraethylorthosilicate as a binder. Therefore, the elemental analysis of GO–C shows the presence of Na and Si-atoms in the resulted EDS analysis, see Fig. 4. By mixing the adsorbent with the adsorbate solution the %At of C, O, Na and Si will be altering and more S-atom appears in the new EDS which confirm the adsorption of the dyes over the adsorbate. Moreover, the appearance of Cu and Co in the elemental analysis of the composite after contacting with their aqueous solutions by using Oxford energy-dispersive X-ray (EDX) spectrometer provide their capturing with the GO–C composite, Fig. 4.

### Adsorption performance of the GO–C composite

In order to evaluate the adsorption efficiency of the GO–C and according to the nature of modified active groups used, four cationic species were select two of them related to organic dyes (MB & CV) and the other inorganic species (Cu^2+^ & Co^2+^). This is due to cover the most main pollutants that results from the industrial activities.

#### Effect of contact time on the removal efficiency

**Figure 5:**
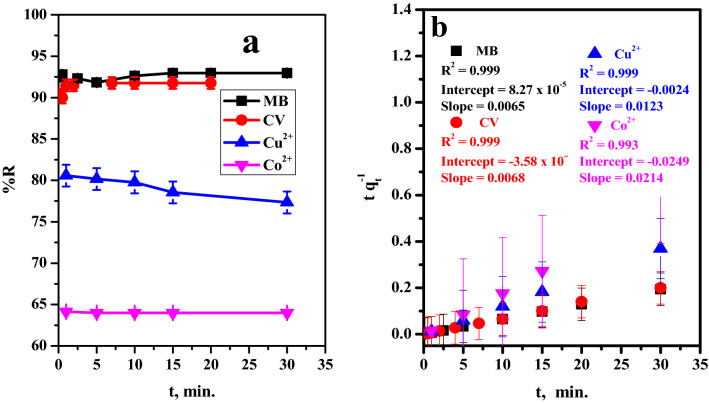
Effect of (**a**) contact time on removal percent, (**b**) Pseudo second-order plot of MB-dye ([[MB] = 20 ppm, Dose = 6 mg, v = 50 ml, pH = 7, T = 30 °C) and CV-dye ([CV] = 20 ppm, Dose = 6 mg, v = 50 ml, pH = 7, T = 30 °C), Cu^2+^ ([Cu^2+^] = 50 mg l^−1^, Dose = 2.4 mg, v = 5 ml, pH = 5, T = 25 °C)and Co^2+^ ([Co^2+^] = 50 mg l^−1^, Dose = 2.4 mg, v = 5 ml, pH = 6, T = 25 °C) from aqueous media.

The effect of contact time that combine the adsorbent and the hazard species consider a vital role in the decontamination efficiency of the pollutant from aqueous solution that it affected on the applicable ability of the prepared materials. Batch adsorptions were studied by stirring the aqueous dye (MB or CV) or heavy metal (Cu^2+^ or Co^2+^) solution with adsorbent for time interval (0.16–30 min). The relation between the contact time and the removal percent was explored in (Fig. 5a). The results showed that, the adsorption kinetics was highly fast even at early adsorption stages. This behavior may be attributed to the high affinity of active function groups (–COO^−^Na^+^) of the citrate towards the cationic pollutant species.

##### Adsorption kinetics

The studied of adsorption rate is required to design a suitable adsorption system, therefore, pseudo first order and pseudo second order were utilized. The adsorption of the pollutants over the GO–C show rapid equilibrium, therefore, the Pseudo first order kinetic model will not be involved. The linear relationship of the used pseudo second order model (see Table S3) presented in (Fig. 5b), and the experimental and calculated data obtained were listed in Table S4. The adsorption rate of the pollutant molecules (MB, CV, Cu^2+^, Co^2+^) over the GO–C show relative stability over all the investigated time, therefore, the pseudo first order was not investigated. On the other hand, at using the pseudo second order the relation coefficient (R^2^) of the hazardous were > 0.99. Moreover, the calculated adsorption capacity related to the adsorbed molecules were very closer to their values obtained from the experiments. This proves that, the pseudo second order best fitted the experimental data.

#### Effect of initial concentration on the removal efficiency

**Figure 6:**
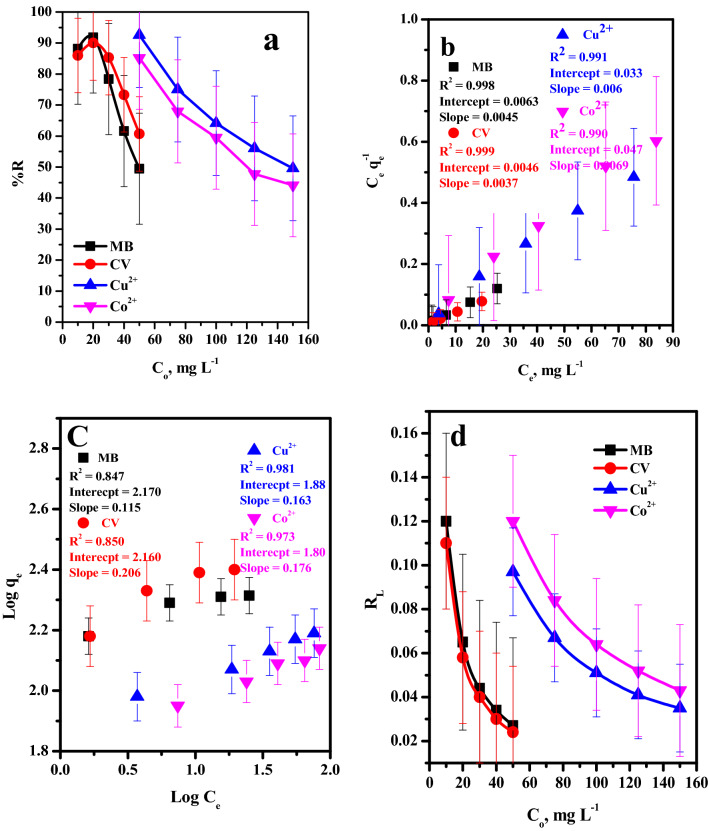
Effect of (**a**) dye concentration on removal percent, (**b**) Langmuir isotherm plot, (**c**) Freundlich isotherm plot (**d**) R_L_ of MB-dye (t = 5 min, Dose = 6 mg, v = 50 ml, pH = 7, T = 30 °C) and CV-dye (t = 7 min, Dose = 6 mg, v = 50 ml, pH = 7, T = 30 °C) Cu^2+^ (t = 1 min, Dose = 2.4 mg, v = 5 ml, pH = 6, T = 25 °C) and Co^2+^ (t = 1 min, Dose = 2.4 mg, v = 5 ml, pH = 8, T = 25 °C) form pure water.

The impact of initial concentration on the removal performance of the different cationic species, MB, CV, Cu^2+^, Co^2+^ by GO–C were investigated in the range (10–50 mg L^−1^) for MB and CV and (50–150 mg L^−1^) for Cu^2+^ and Co^2+^, as explored in Fig. 6a. It was seen that the elimination efficiency of the two dyes decrease significantly 88–49% (MB) and 86–60% (CV)as the initial dye concentration increase in rang (10–50 mg L^−1^) for both dyes, 92.54–49.59% (Cu^2+^) and 85.21–44.12% (Co^2+^) as the initial heavy metal ion concentration increase in range (50–150 mg L^−1^) for both metal ions. This is referred to, at low pollutants concentration, the number of the adsorbent active sites were sufficient for adsorb the pollutant species. As the initial adsorbate concentration increase the number of available active site will be decrease compared to the number of adsorbate species.

##### Adsorption isotherms

Adsorption isotherms are required to ascribe the relation among the quantity of the adsorbed species and its concentration in the aqueous solution at reaching equilibrium at constant temperature. The parameters calculated from various models supply interested information about the adsorption mechanism, the surface character and affinity of the adsorbent. The most used models named as Langmuir and Freundlich models, see Table S5.

The graphs of C_e_ versus C_e_/q_e_ and log C_e_ versus log q_e_, Fig. 6b,c, respectively. The evaluated parameters of the models and correlation coefficient (R^2^) for the alter adsorptions species are recorded in Table 2. According to R^2^ values, the Langmuir equation shows a better fit than the Freundlich with superior adsorption performance for MB (222.22 mg g^−1^), CV (270.27 mg g^−1^), Cu^2+^ (163.40 mg g^−1^) and Co^2+^ (145.35 mg g^−1^) Table 2. Consequently, the significant character of the Langmuir isotherm can be expressed in terms of dimensionless separation parameter, R_L_, Fig. 6d, which is term of the isotherm shape that indicates if the adsorption system is favorable or unfavorable. R_L_ is defined as (Eq. 1):

1 $$ \,R_{L} = \frac{1}{{1 + bC_{o} }}\,\,\,\,\,\,\,\, $$

where, b is the Langmuir constant. In this work R_L_ values located among 0 and 1 (0.12 for MB, 0.11 for CV, 0.09 for Cu^2+^ and 0.12 for Co^2+^) as indicated in Table 2 which demonstrated applicable adsorption process.

**Table 2:** Langmuir and Freundlich constants for adsorption of MB-dye (t = 5 min, Dose = 6 mg, v = 50 ml, pH = 7, T = 30 °C) and CV-dye (t = 7 min, Dose = 6 mg, v = 50 mL, pH = 7, T = 30 °C), Cu^2+^ (t = 1 min, Dose = 2.4 mg, v = 5 ml, pH = 6, T = 25 °C) and Co^2+^ (t = 1 min, Dose = 2.4 mg, v = 5 ml, pH = 8, T = 25 °C) form aqueous solution.

Dye	Langmuir isotherm model				Freundlich isotherm model		
	q_o_ (mg/g)	b (l/mg)	R_L_	R^2^	1/n	K_f_ (mg/g)	R^2^
MB	222.22	0.71	0.12	0.998	0.115	147.91	0.847
CV	270.27	0.80	0.11	0.999	0.206	144.54	0.850
Cu^2+^	163.40	0.185	0.097	0.999	0.16	77.40	0.980
Co^2+^	145.35	0.146	0.12	0.989	0.18	62.72	0.954

Moreover, Freundlich model is property by $\frac{1}{n}$ heterogeneity factor; where, the sites on the surface were not have the same binding energy. The values of 1/n between 0.1 < $\frac{1}{n}\hspace{0.17em}$< 1.0 that explored suitable adsorption of the pollutants onto the adsorbent.

#### Effect of adsorbent dosage on the removal efficiency

In order to study the effect of adsorbent GO–C on the removal efficiency (%R) of various pollutant species, various amounts of adsorbent (4–18 mg) for the dye and (2.4–8.4 mg) in case of heavy metal ion were blended with the wasted aqueous solutions and the obtained results were plotted in Fig. S4a. The obtained data showed that the increase in the quantity of the adsorbent dose (4–8 mg) (for the dyes) and (2.4–6 mg) for metal ions was followed with significant increase in the removal percent for all species. Further increase in adsorbent dose amount presented equilibrium adsorption rate. This may be ascribed as; at low adsorbent dose the GO–C layers were completely separated and the active sites fully exposed to the contaminated solution which enhance the adsorption efficiency see SEM images. On the other hand, the increase in the adsorbent dose will cause GO–C agglomeration which will lead to obscure most of the active sites available for adsorption of the adsorbed species (Fig. S4b,c).

#### Effect of aqueous solution pH on the removal efficiency

**Figure 7:**
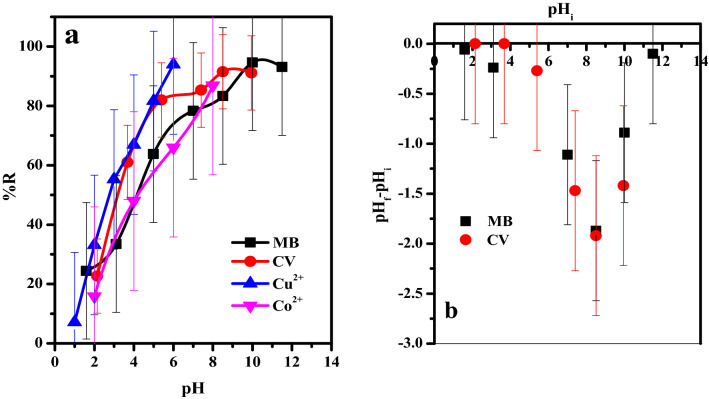
(**a**) Effect of the initial solution pH on The removal percentage (% R) from aqueous solution, (**b**) pH_ZPC_ of the GO–C adsorbent of MB-dye (t = 5 min, [MB] = 30 ppm, dose = 6 mg, v = 50 ml, T = 30 °C) and CV-dye (t = 7 min, [CV] = 30 ppm, dose = 6 mg, v = 50 ml, T = 30 °C), Cu^2+^([Cu^2+^] = 50 mg l^−1^, Dose = 2.4 mg, v = 5 ml, T = 25 °C) and Co^2+^(t = 1 min, [Co^2+^] = 50 mg l^−1^, Dose = 2.4 mg, v = 5 ml, T = 25 °C) form aqueous media.

The pH of the adsorption solution is considered an interested condition in the uptake process. Where, it can regular the mechanism for elimination of pollutants since the pH value can impacted on the active sites located on the adsorbate surface. The influence of the solution pH on the uptake percent of the GO–C was plotted in (Fig. 7a). It was clearly seen that, the adsorption percent increase sharply ((24–94% (MB), 22–91% (CV), 7–94% (Cu^2+^) and 16–87% (Co^2+^)) with further increase in the pH value in the range 1.6–11.5 (MB), 2.15–9.95 (CV), 1–6 (Cu^2+^) and 2–8 (Co^2+^).

On focus, the point of zero charge (PH_ZPC_) is known as the pH value at which the net charge of the adsorbent, due to H^+^ and OH^−^ ions interaction with functional groups located on its surface, is null. Hence, we can determine of PH_ZPC_ by plotting ΔpH (pH_f_–pH_i_) against pH_i_, where pH_i_, the initial pH value of the solution and pH_f_, the pH of the solution after the treatment process. The data obtained from the experiments were plotted in Fig. 7b. The results obtained showed that the composite (GO–C) is completely ionized even at very low pH (1.6). This regime is attributed to the GO was modified with tri-sodium citrate which has three sodium carboxylate (–COO^−^ Na^+^) groups, these groups are already ionized. Therefore, the presentation of the GO–C in the dye solution will increase the negativity of the adsorption solution.

Based on the pervious information, the mechanism of adsorption of the cationic species (MB, CV, Cu^2+^ and Cu^2+^) could be suggested. At low pH value H^+^-ion compete the dye species for the available active sites (–COO^−^ Na^+^) on the adsorbent and form (–COOH) and so the affinity of the adsorbent towards the cationic species decreases. With increase in the solution pH value the concentration of H^+^-ion decrease and the competition for the active sites will be minimized and so the adsorption percent of the cationic species enhanced. Moreover, the further increase in the pH over 10 (MB) and 8.5 (CV) the removal percent of the dye tend to be stable. This may be attributed to at high alkaline solution the activated function groups (–COO^−^ Na^+^) introduce over the adsorbent suffer from low ionization by the common ion effect due to concentration of Na^+^-ion of the solution^33^. Moreover, based on the EDS analysis, Fig. 4, of the GO–C showed the presence of Na^+^ which related to the (COO^−^Na^+^) groups of the citrate molecules. After mixing the GO–C composite with the aqueous solutions of MB, CV, Cu^2+^ and Co^2+^, the %At of the Na^+^ will highly reduced or almost disappear which indicated that the pollutant species adsorbed via cationic exchange mechanism.

#### The effect of the aqueous solution temperature on the removal percent

To evaluate the impact of the temperature on the adsorption behavior of the adsorbent, the adsorption processes were carried out at different temperature values ranged from 30 to 95 °C for the dye and 25–60 °C for the heavy metal ions. The relation between the temperature and the adsorption performance (% R) were plotted in Fig. S5a. The data obtained revealed that, there was little variations in the removal percent of the two dyes as the temperature differ from 30 to 95 °C. On the other hand, there is a slow increase in the up-take percent of the metal ions with further increase in solution temperature from 25 to 60 °C.

##### Thermodynamic studies

The standard thermodynamic parameters of the sorption process represented through Gibbs free energy, ΔG°, Enthalpy, ΔH°, and Entropy, ΔS°. These represent the main thermodynamic function to assess sorption reaction. Vant Hoff equation was employed to evaluate Gibbs free energy as in Table S6.

The enthalpy change, ΔH° and the entropy, ΔS° were obtained from the equation presented in Table S6. The plot of ln K_d_ versus 1/T, a straight line was obtained as illustrated in Fig. S5b. From the slope, the value of ΔH° was calculated at 25 °C as shown in Table S7. While Entropy change, ΔS° is obtained from the intercept as shown in Table S7. It is clear that the sorption of Cu and Co ions is endothermic and spontaneous reaction. Further, the positive entropy changes indicate the randomness of the adsorption process.

#### Regeneration and reusability

**Figure 8:**
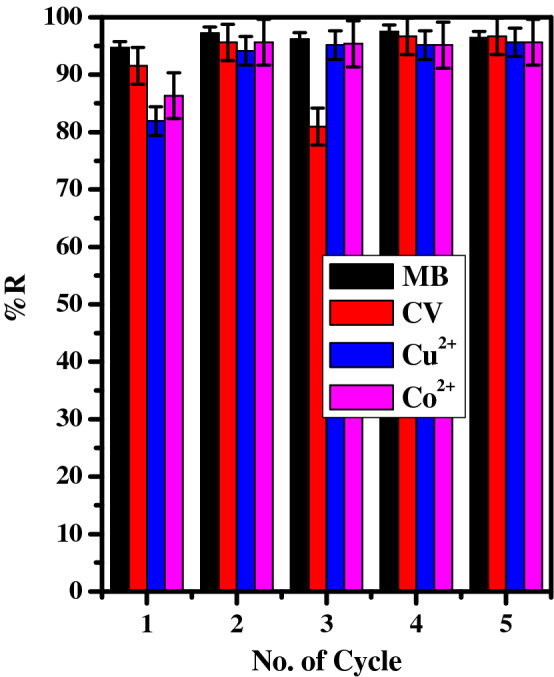
The effect of the number of the re-use cycles of the GO–C on the removal percentage of MB-dye (t = 5 min, [MB] = 30 ppm, dose = 6 mg, v = 50 ml, pH = 7), CV-dye (t = 5 min, [CV] = 30 ppm, dose = 4 mg, v = 50 ml, pH = 7), Cu^2+^ ([Cu] = 100 mg l^−1^, Dose = 6 mg, v = 5 ml, pH = 6, T = 25 °C) and Co^2+^ ([Co] = 100 mg l^−1^, Dose = 6 mg, v = 5 ml, pH = 8, T = 25 °C) form aqueous media. The regenerated solution (10 ml (10%HCl) + 5 mL (1 M NaOH)).

Five adsorption–desorption runs were carried out for test the reusability performance of the adsorbent, as indicated in Fig. 8. The results indicate that, there was an increase in the adsorption percent of the all pollutants upon regeneration and reuse over five cycles. This may be attributed to the using of the NaOH will be activated extra function groups located on the GO-surface, refer Fig. 1. Hence, the number of available active site increase, consequently, the affinity towards the cationic pollutants will be performed, thereafter, the adsorption percent will be enhanced. Moreover, the regeneration-reusability function of GO–C, will reduce the applicable cost.

#### Selective adsorption for cationic dyes and practical application

**Figure 9:**
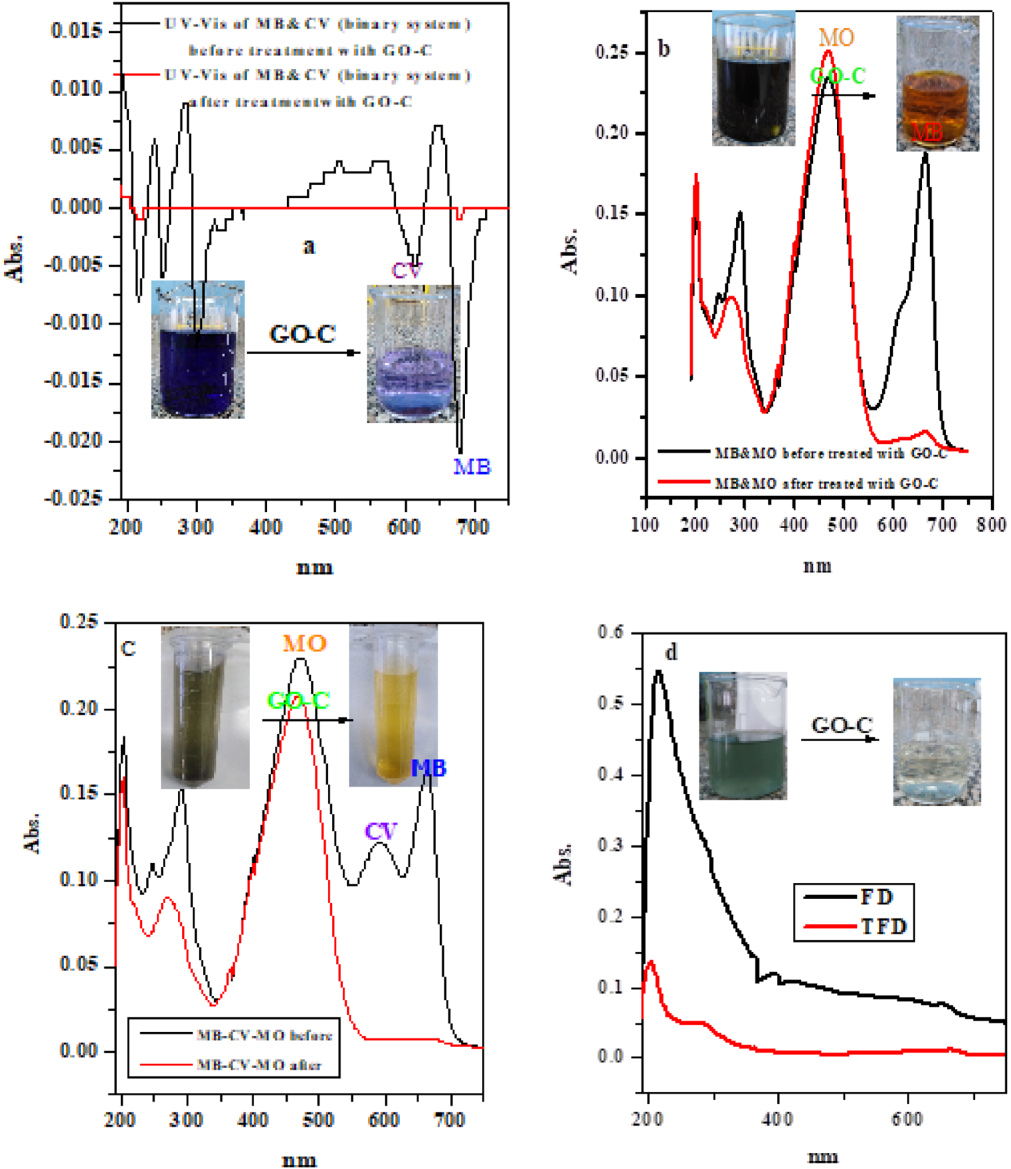
UV–Vis spectra of; (**a**) (MB–CV) binary system solution before and after treated with GO–C composite, (**b**) (MB–MO) binary system solution before and after treated with GO–C composite, (**c**) (MB–CV–MO) trinary system solution before and after treated with GO–C composite and (**d**) factory real sample (FD) and treated factory real sample (TFD).

Based on the nature of the surface functionalized group (–COO^−^Na^+^) and high adsorption performance of the GO–C, it was suggested that the GO–C have ability to select adsorption of cationic dyes. In this regard, the adsorption of cationic dyes (MB & CV) and (MB & MO) from binary system and (MB, CV & MO) from trinary system were investigated and the findings were monitored visually and with the UV–Vis spectroscopy, as present in Fig. 9a–c. The results showed that, the GO–C composite can completely remove the MB and CV dyes (binary system) from the aqueous solution, and this result confirmed from the completely diminished of the MB and CV peaks at the end of the adsorption process, see Fig. 9a. Moreover, upon mixing of cationic dyes (MB or (MB&CV)) with the anionic dye (MO-dye), they yielded solution with green color, as indicated in Fig. 9b,c. By treating the previous solutions with the GO–C composite the color of the blended solution turned into orang, crossponding to the color of MO-dye, and more, the UV–Vis spectra indicated the completely disappearance of the peaks related to the MB and CV, refer Fig. 9b,c, after the treatment process. These results provided that the GO–C composite can adsorb cationic dyes from binary system, and select adsorption of singlet and binary cationic dyes mixed with anionic dye. Moreover, the GO–C was applied for cleaning real wastewater sample as shown in Fig. 9d as demonstrated from the Fig. 8d, the GO–C composite was success in purification of the treated real sample. These results indicates that the as-prepared GO–C nanocomposite could be applied for treatment of the industrial effluents.

#### Comparison with other adsorbent material

Comparing with several materials that listed in the literature^17,34–57^, the adsorption performance of GO–C towards various cationic adsorbent (MB, CV, Cu^2+^, Co^2+^) was highly enhanced, see Table 3. Therefore, we can conclude that the GO–C can be consider as a superior adsorbent due to its easy, low-cost synthesis process and excellent affinity for wide range pollutants.

**Table 3:** Adsorption capacities of various adsorbents of MB, CV, Cu^2+^, Co^2+^.

Adsorbate	Adsorbent	q° (mg/g)	Refs.
MB	Activated charcoal	76.92	^34^
	Graphene oxide-activated carbon	147	^35^
	Activated serpentinemineral decorated with magnetic nanoparticles	162	^36^
	AFe	53.79	^37^
	Modified clinoptilolite	52	^38^
	GO/WCE	57.60	^39^
	CMC-Alg/GO hydrogel beads	78.5	^40^
	GO–C	222.22	This work
CV	Polyacrylonitrile/β-cyclodextrin/graphene oxide nanofibers composite	15.84	^41^
	Activated charcoal	33.33	^34^
	Graphene oxide-activated carbon	70	^35^
	Palm kernel shell-derived biochar	24.45	^42^
	Natural Fly ash modified with calcium oxide	38.75	^43^
	Mesocellular foam (MCF) silica molecular sieve	6.646	^44^
	ZVI-GAM	172.41	^45^
	GO–C	270.27	This work
Cu^2+^	CGP3 beads	163.4	^46^
	Gelatin-glutaraldehyde-PEI	61.2	^47^
	Carboxylatedcellulose nanofibers	74.2	^48^
	GO	117.5	^49^
	GO–NH2	26.25	^17^
	Grafting polyacrylamide onto G	121.542	^50^
	GO–C	163.4	This work
Co^2+^	GO	21.28	^51^
	mPal-GO	16.9	^52^
	GO	79.36	^53^
	AC	90.36	^54^
	Clearing nut seed powder	4.245	^55^
	Carboxymethyl chitosan beads	46.25	^56^
	Cyanoethyl modified magnetic chitosan	17.92	^57^
	GO–C	145.35	This work

## Conclusion

Citrate modified graphene oxide (GO–C) was simply prepared and investigated to eliminate dyes and heavy metals from aqueous solution. The structure of the composite was characterized employing SEM, OM, FTIR, Raman, EDS. According to the TGA analysis, the –OH groups located on the GO basal plane share in the modification step. This manner highly affected on the thermal properties of the GO–C composite compared with the GO. Moreover, the adsorption of the dye by the GO–C was found also enhance the thermal stability of the GO–C–MB complex. Attributed to the modification of the GO with sodium citrate, the GO–C showed a high rapid kinetics and an excellent affinity for MB, CV, Cu^2+^ and Co^2+^, the crossponding adsorption capacity performance according to Langmuir were 222.22, 270.27, 163.4 and 145.35 mg g^−1^ under a single system which were larger than presented in the literature. The composite shows additional properties, that the it can selective adsorption of the cationic dyes in singlet and binary system from aqueous solution. Additionally, the GO–C regenerated/reused over five cycle times with increasing in the adsorption percent than related to the pristine composite.

## Supplementary Information

Supplementary Information.

## Data Availability

All data generated or analyzed during this study are included in this published article and its supplementary information files.
