# Supplementary material for: Superior adsorption performance of citrate modified graphene oxide as nano material for removal organic and inorganic pollutants from aqueous solution
Source: Sci Rep. 2022 Jun 2;12:9204. doi: 10.1038/s41598-022-13111-6 (PMC9163102; doi:10.1038/s41598-022-13111-6)
Supplement: Supplementary file 1 — Supplementary Information. [file 41598_2022_13111_MOESM1_ESM.docx]

**Supplementary Information**

**Superior adsorption performance of citrate modified graphene oxide as nano material for removal organic and inorganic pollutants from aqueous solution**

A.I. Abd-Elhamid^a*^, E.M. Abu Elgoud^b^, Sh.Sh. Emam^b^, H.F. Aly^b^

^a^ Composites and Nanostructured Materials Research Department, Advanced Technology and New Materials Research Institute (ATNMRI), City of Scientific Research and Technological Applications (SRTA-City), New Borg Al-Arab 21934, Alexandria, Egypt

^b^Hot Laboratories Center, Egyptian Atomic Energy Authority, 13759, Egypt

^*^Corresponding author, email: [ahm_ch_ibr@yahoo.com](mailto:ahm_ch_ibr@yahoo.com)

1. **Experiment**

**1.1. Materials and instrumentation**

The chemicals required were of analytical grade and utilized without further purification. H_2_SO_4_ (95–97%, Riedel deHaen), H_2_O_2_ (36%, Pharaohs Trading and Import), HCl (30%, El Salam for Chemical Industries), KMnO_4_ (99%, Long live), and graphite (200mesh, 99.99%, Alpha Aesar). Methylene Blue (MB) (Sigma-Aldrich), Crystal Violet (CV), Methyl Orang (MO) (Sigma-Aldrich), CuCl_2_ (99%, Sigma-Aldrich), Co(NO_3_)_2_·6H_2_O (99%, Sigma-Aldrich), tri-sodium citrate (Sigma-Aldrich), TEOS (99%, Across), ethanol absolute (Sigma-Aldrich).

Analytical balance (CP 2245, Sartorius, USA.), Hot plate stirrer (IKA, C-MAG HS7, IKA®-Werke GmbH & Co. KG, Germany), pH meter (3510, Genway), Hot plate stirrer (SB 162, Stuart, UK.), Centrifuge, (Mikro 220R, Hettich, UK.), UV/Vis. Spectrophotometer-Double beam (T80+, PG instruments Ltd., UK.).

**1.2. Characterization**

The prepared material was characterized by Scanning Electron Microscope (SEM) (JEOL GSM-6610LV. Japan) with additional EDS unit, Light Optical Microscopy (LOM) (BX61, Olympus, Japan), Thermo-Gravimetric Analysis (TGA, Shimadzu Thermal Gravimetric Analysis (TGA)—50, Japan). Elemental composition of Cu (II) and Co (II) sorption on GO-C was displayed by an Oxford energy-dispersive X-ray (EDX) spectrometer (Oxford Link ISIS, Japan). Fourier Transmission Infra-Red Spectroscopy (FT-IR) (8400s, Shimadzu, Japan) covered the range from 400 to 4000 cm^−1^ and Raman Spectroscopy (Bruker, Senterra II, Germany).

**1.3. Batch Adsorption of organic pollutants**

In this experiment a 50 ml of definite concentration of the dye solution was mixed with the GO-C over a time range (0.16 – 30 min.), the initial dye concentrations of (10-50 mg L^-1^) were used. Various quantity of adsorbents (4-18 mg) was utilized. The of the dye solutions pH values were investigated from 1.6 to 11.5 for MB and 2.15 to 9.95 for CV. The temperature in rang 30 – 95^o^C were applied for both dyes. 2 ml of the treated dye solutions was isolated and centrifuged. Thereafter, 0.5 ml was diluted to 5 ml by distill water and the residual dye concentration was detected at 662 nm for MB and 590 nm for CV using UV-Vis spectrophotometer.

The dye removal percent (%R) is defined as:

$\% R=\frac{C_{o}-C_{t}}{C_{t}}\times100$ (1)

Where, C_o_ and C_t_ are the concentration of the dye species at time zero and t, respectively.

**1.4. Batch Adsorption of inorganic pollutants**

Initially, Cu (II) and Co (II) stock solutions (1.0 g/L) were synthesized by adding a definite weight of CuCl_2_ and Co (NO_3_)_2_ · 6H_2_O in a known volume of bidistilled water. Further required concentrations of the investigated metal ions were prepared by dilution. A batch sorption studies were performed in order to test the parameters that affect the Cu (II) and Co (II) adsorption, such as contact time, pH, v/m, initial Cu (II) and Co (II) concentration, and temperature, where the optimized conditions were determined. The concentration metal maintained in the solution was detected spectrophotometrically using Shimadzu double beam spectrophotometer by complexing with 4-(pyridyl-2-azo) resorcinol (PAR)^1^. The %R of metal retained in the GO-C, was calculated using Eq. 1.

The practical experiments were performed three times for each experiment, and then the standard deviation values were calculated from the following equation S1:

$S=\sqrt{\frac{\sum(X-\bar{x})^{2}}{n-1}}$ S1

Where, S is standard deviation, X each value, x̅ sample mean and n number of values in the sample.

Then, all the adsorption figures and statistical error calculations are used for evaluation of the suitability of model good fits were investigated with Origin 8.5

**1.5. Regeneration and reusability**

After adsorption, the pollutants taken by adsorbent were eluted by desorption-regeneration using solutions of (10 mL (10 % HCl), 5 mL distilled H_2_O, 5 mL (1.0 M NaOH) and finally, washed by 5 mL distilled H_2_O) for the next use.

**1.6. Selective adsorption for cationic dyes and practical application**

To study the selective adsorption affinity of GO-C towards the cationic dye, firstly, 200 mL of binary system of cationic (MB-CV) dye solutions with the same concentration (10 mg L^−1^) was stirred with 12 mg of GO-C over five minutes. Thereafter, the UV–vis spectra of the mixed solution were measured before and after adsorption process. Secondly, binary system of (MB (cationic species)-MO (anionic species)) dye solution (200 mL) was prepared by blending equal concentration (10 mg L^-1^) and contacted with 12 mg of GO-C composite for (5 min.). At the end of the experiments the UV–vis spectra assessed the mixed solution before and after adsorption process. Finally, trinary system of (MB&CV (cationic species)-MO (anionic species)) dye solution (200 mL) was prepared by combine similar concentrations of the three dyes (10 mg L^-1^) and further treated with 12 mg GO-C for 5 minutes. Afterwards, the UV–vis spectra was employed to analysis of the mixed solution before and after the treatment process.

**3. Results and Discussion**

**3.1. Figures**

| **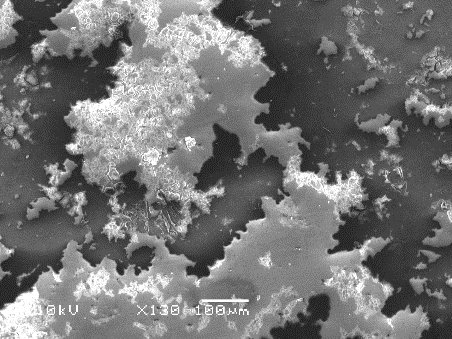 GO-C-MB** | **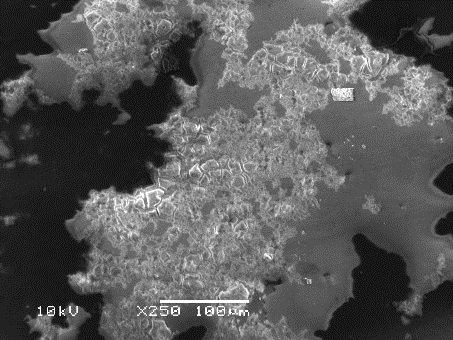** | **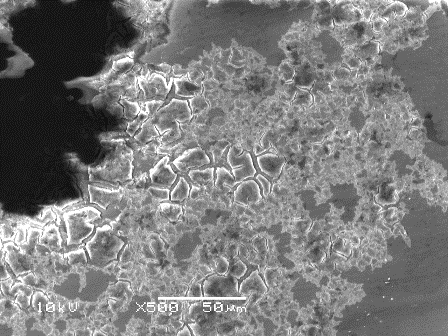** |
| --- | --- | --- |
| **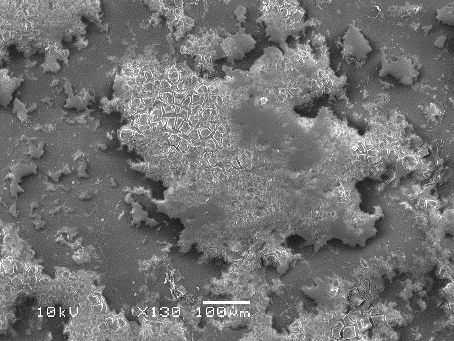 GO-C-CV** | **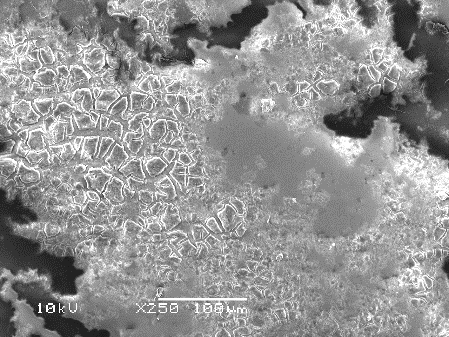** | **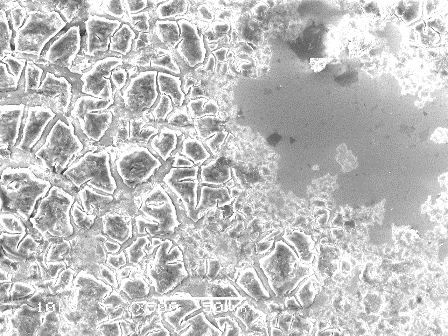** |

**Fig. S1.** SEM images for GO-C-MB and GO-C-CV complex.

| **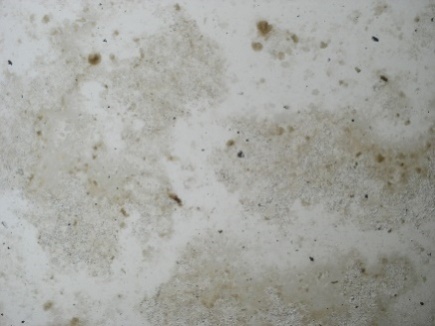 GO-C 5X** | | **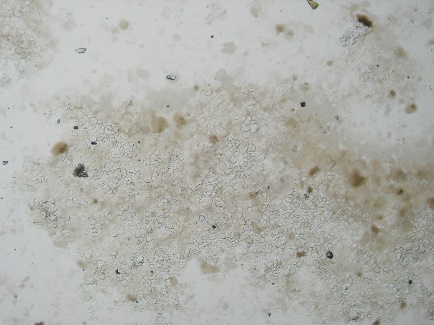 10X** | **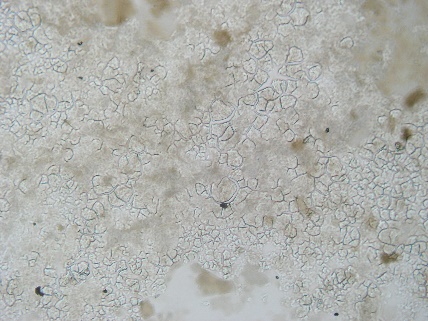 20X** |  |
| --- | --- | --- | --- | --- |
| **GO-C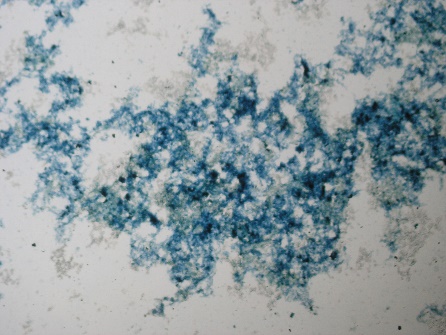-MB 5X** | **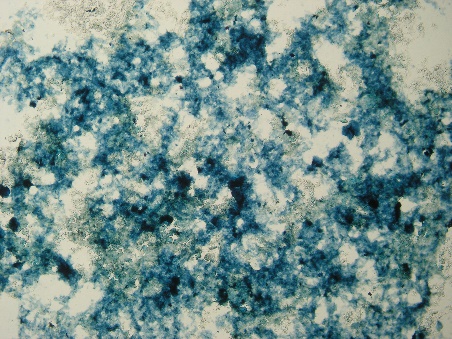 10X** | | **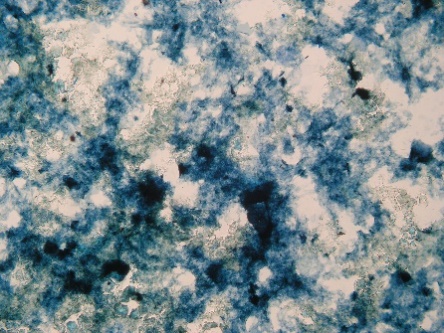 20X** | |
| **GO-C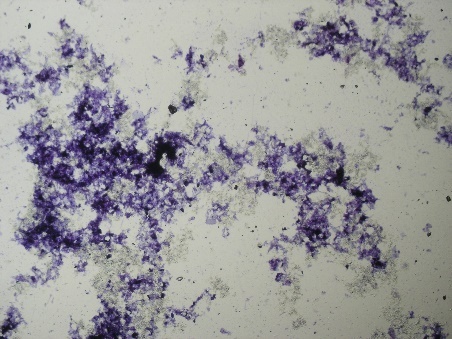-CV 5X** | **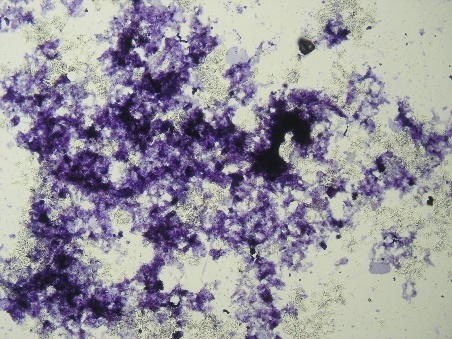 10X** | | **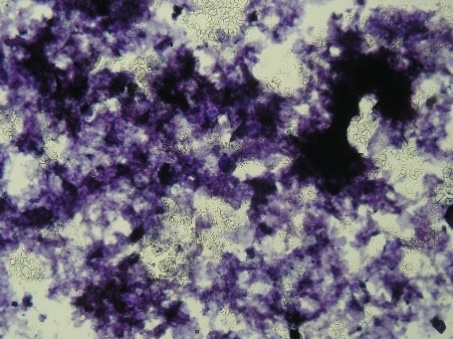 20X** | |

**Fig. S2.** OM images for GO-C, GO-C-MB and GO-C-CV complex.

**Fig. S3.** Raman spectrum for GO, GO-C composite, GO-C-MB complex and GO-C-CV complex.

|  | 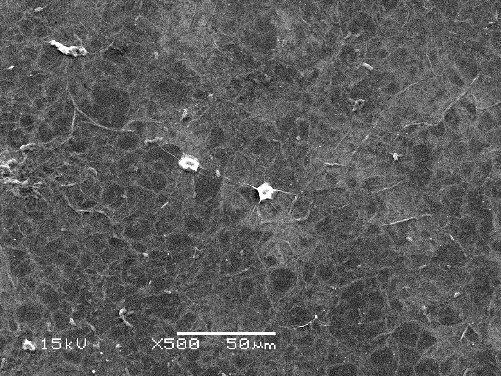b |
| --- | --- |
|  | 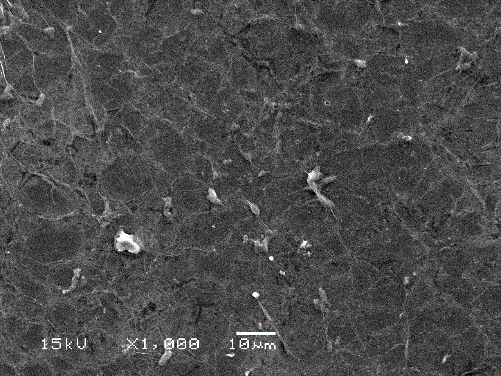c |

**Fig. S4.** Effect of GO-C dose/50ml dye solution on removal percent of MB-dye (t = 5 min, [MB] = 30 ppm, v= 50 mL, pH = 7, T = 30 ºC) and CV-dye (t = 7 min, [CV] = 30 ppm, v= 50 mL, pH = 7, T = 30 ºC) Cu^2+^(t = 1 min, [Cu^2+^] = 100 mg L^-1^, v= 5 mL, pH = 5, T = 25ºC) and Co^2+^(t = 1 min, [Co^2+^] = 100 mg L^-1^, v= 5 mL, pH = 6, T = 25 ºC) form aqueous media

|  |  |
| --- | --- |

**Fig. S5.** Effect of temperature on the removal percent of MB-dye (t = 5 min, [MB] = 30 ppm, dose = 6 mg, v= 50 mL, pH= 7) and CV-dye (t = 5 min, [CV] = 30 ppm, dose = 4 mg, v= 50 mL, pH= 7), Cu^2+^([Cu^2+^] = 100 mg L^-1^, Dose = 6 mg, v= 5 mL, pH = 6, T = 25 ºC) and Co^2+^([Co^2+^] = 100 mg L^-1^, Dose = 6 mg, v= 5 mL, pH = 8, T = 25 ºC) form aqueous media.

**3.2. Tables**

**Table S1.** The D and G-band positions, I_D_/I_G_ ratios and FWHMs.

| Sample | D-band peak | | |  | G-band peak | | I_D_ /I_G_ |
| --- | --- | --- | --- | --- | --- | --- | --- |
|  | Raman shift (cm^-1^) | | FWHM (cm^-1^) |  | Raman shift (cm^-1^) | FWHM(cm^-1^) |  |
| GO | 1352 | 150 | |  | 1598 | 112 | 1.34 |
| GO-C | 1351 | 112 | |  | 1594 | 88 | 1.27 |
| GO-C-MB | 1339 | 87 | |  | 1588 | 63 | 1.38 |
| GO-C-CV | 1357 | 112 | |  | 1587 | 75 | 1.49 |

**Table S2.** TGA analysis of GO, GO-C and GO-C-MB.

| **GO** | | | **GO-C** | | | **GO-C-MB** | | |
| --- | --- | --- | --- | --- | --- | --- | --- | --- |
| **T, ^o^C** | **wt loss %** | **Degradable Species** | **T, ^o^C** | **wt loss %** | **Degradable Species** | **T, ^o^C** | **wt loss %** | **Degradable Species** |
| 28-89 | 19.60 | moisture | 27-92 | 8.92 | moisture | 19-82 | 8.79 | moisture |
| 89-158 | 4.07 | H_2_O | 92-139 | 1.78 | H_2_O | 82-153 | 3.00 | H_2_O |
| 158-214 | 20.50 | -OH | 139-194 | 6.05 | -OH | 153-201 | 6.15 | -OH |
| 214-320 | 8.40 | -COOH | 194-324 | 4.15 | -COOH | 201-470 | 7.20 | -COOH/MB |
| 320-600 | 5.30 | Ash | 324-474 | 14.62 | Citrate | 470-668 | 20.70 | Citrate/MB |
|  |  |  | 474-800 | 1.00 | Ash | 668-800 | 0.50 | Ash |

**Table S3.** Linear equation for of the adsorption kinetic model^2^.

| model | Linear form | Graph |
| --- | --- | --- |
| Pseudo Second Order | $\frac{t}{q_{t}}=\frac{1}{K_{2}q_{e}^{2}}+\frac{t}{q_{e}}$ | t vs t/q_t_ |

**Table S4.**  Pseudo-ﬁrst-order and pseudo-second-order kinetic model parameters for adsorption of MB-MB-dye([[MB] = 20 mg L^-1^, Dose = 6 mg, v= 50 mL, pH = 7, T = 30 ºC), CV-dye ([CV] = 20 mg L^-1^, Dose = 6 mg, v= 50 mL, pH = 7, T = 30 ºC), Cu^2+^([Cu^2+^] = 50 mg L^-1^, Dose = 2.4 mg, v= 5 mL, pH = 5, T = 25 ºC) and Co^2+^([Co^2+^] = 50 mg L^-1^, Dose = 2.4 mg, v= 5 mL, pH = 6, T = 25 ºC) from aqueous media.

| Dye | q_e exp_ (mg /g) |  | Second-order kinetic parameter | | |
| --- | --- | --- | --- | --- | --- |
|  |  |  | K_2_ (g mg^-1^min^-1^) | q_ecal_(mg /g) | R^2^ |
| MB | 154.92 |  | 0.51 | 153.85 | 0.999 |
| CV | 149.17 |  | -0.13 | 147.1 | 0.999 |
| Cu^2+^ | 81.20 |  | -0.033 | 80.78 | 0.998 |
| Co^2+^ | 47.46 |  | -0.018 | 46.73 | 0.993 |

**Table S5**. Linear equations and graphs of the adsorption isotherms^3, 4^.

| Isotherm | Linear form | Graph |
| --- | --- | --- |
| Langmuir isotherm | $\frac{C_{e}}{q_{e}}=\left( \frac{1}{Q_{o}} \right)b+\left( \frac{1}{Q_{o}} \right)C_{e}$ | C_e_ vs C_e_/q_e_ |
| Freundlich isotherms | $\log q_{e}= \log K_{f} + \frac{1}{n} \log C_{e}$ | Log C_e_ vs Log q_e_ |

**Table S6**. Thermodynamic equations and graphs of the adsorption process^5^.

| Isotherm | Linear form | Graph |
| --- | --- | --- |
| Vant Hoff | $\Delta G^{\circ}= -RTlnK_{d}$ | ΔG vs Ln K_d_ |
|  | $\ln K_{d}=\frac{\Delta S^{o}}{R}+\frac{-\Delta H^{o}}{R}\frac{1}{T}$ | Ln K_d_ vs 1/T |

**Table S7.** Standard thermodynamic parameters for **Cu^2+^** and **Co^2+^** removal from aqueous solution utilizing GO-C composite.

| **T (K)** | **ΔG (kJ/mole)** | | **ΔH(kJ/mole)** | | **ΔS (J/mole/K)** | |
| --- | --- | --- | --- | --- | --- | --- |
|  | **Cu^2+^** | **Co^2+^** | **Cu^2+^** | **Co^2+^** | **Cu^2+^** | **Co^2+^** |
| **298** | -23.46 | -24.08 | 6.16 | 11.23 | 99.46 | 118.32 |
| **308** | -24.48 | -25.17 |  |  |  |  |
| **318** | -25.46 | -26.28 |  |  |  |  |
| **328** | -26.51 | -27.62 |  |  |  |  |
| **338** | -27.43 | -28.80 |  |  |  |  |

1. Z. Marczenko. Spectrophotometric determination of elements. New York: John Wiley and Sons, Inc (1976).
2. Y.S. Ho and G. McKay, The sorption of lead (II) ions on peat, Water Res., 33 (1999) 578 – 584.
3. I. Langmuir, The constitution and fundamental properties of solids and liquids. Part I. Solids, J. Am. Chem. Soc. 38 (11) (1916) 2221–2295.
4. H. Freundlich, Over the adsorption in solution, J. Phys. Chem. 57 (1906) 385–470.
5. A.A. El-Bayaa, N.A. Badawy, A.M. Gamal, I.H. Zidan, A.R. Mowafy, Purification of wet process phosphoric acid by decreasing iron and uranium using white silica sand. J. Hazard. Mater. 190 (2011) 324–329.
